# Motor Imagery EEG Signal Recognition Using Deep Convolution Neural Network

**DOI:** 10.3389/fnins.2021.655599

**Published:** 2021-03-25

**Authors:** Xiongliang Xiao, Yuee Fang

**Affiliations:** ^1^College of Electronic Science and Engineering, Hunan University of Information Technology, Changsha, China; ^2^College of Electric Power Engineering, Hunan Polytechnic of Water Resources and Electric Power, Changsha, China

**Keywords:** EEG signal, motor imagination, deep convolutional neural network, short time fourier transform, continuous morlet wavelet transform, BCI classifier, CSP algorithm

## Abstract

Brain computer interaction (BCI) based on EEG can help patients with limb dyskinesia to carry out daily life and rehabilitation training. However, due to the low signal-to-noise ratio and large individual differences, EEG feature extraction and classification have the problems of low accuracy and efficiency. To solve this problem, this paper proposes a recognition method of motor imagery EEG signal based on deep convolution network. This method firstly aims at the problem of low quality of EEG signal characteristic data, and uses short-time Fourier transform (STFT) and continuous Morlet wavelet transform (CMWT) to preprocess the collected experimental data sets based on time series characteristics. So as to obtain EEG signals that are distinct and have time-frequency characteristics. And based on the improved CNN network model to efficiently recognize EEG signals, to achieve high-quality EEG feature extraction and classification. Further improve the quality of EEG signal feature acquisition, and ensure the high accuracy and precision of EEG signal recognition. Finally, the proposed method is validated based on the BCI competiton dataset and laboratory measured data. Experimental results show that the accuracy of this method for EEG signal recognition is 0.9324, the precision is 0.9653, and the AUC is 0.9464. It shows good practicality and applicability.

## Introduction

Brain-Computer Interface (BCI) is a communication control system established between the brain and external devices (computers or other electronic devices) through signals generated during brain activity (Gao, 2008; Mohamed et al., 2017; Wang et al., 2019). The system does not rely on muscles and nerves other than the brain, and establishes direct communication between the brain and the machine. It is a new and high-end human-computer interaction method.

**FIGURE 1:**
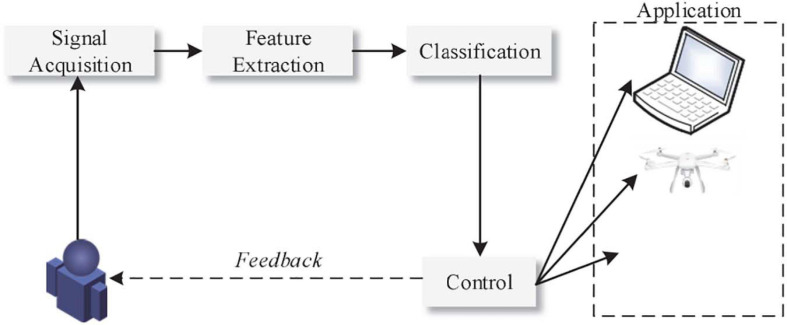
Structure of motor imagery recognition system.

The Motor Imagery Brain-Computer Interface (MI BCI) based on electroencephalogram (EEG) belongs to the category of spontaneous brain-computer interface (Abiri et al., 2018). The purpose of MI BCI is to accurately identify the user’s physical movement intentions, which commonly include imagination of left and right hands, feet, and tongue movements. This is of great significance to the fields of medical rehabilitation, leisure and entertainment (Vasilyev et al., 2017; Minkyu et al., 2018). Figure 1 is a structural diagram of a simple motor imagery recognition system. However, due to the non-stationary, non-linear, low signal-to-noise ratio and other characteristics of EEG signals, there are still many problems to be solved in terms of preprocessing, feature extraction, and multi-mode classification (Yazici et al., 2019). As a result, there are fewer BCI systems that can be practically applied.

The EEG signal is a multi-channel signal, and there is no perfect theory for the role of each channel in classification. For EEG signal identification and classification, the traditional methods are mostly to manually select channels for experiments. In this process, it is possible to lose effective features or introduce unnecessary noise (Duan et al., 2020). At the same time, the EEG signal has its own low signal-to-noise ratio and other characteristics, plus interference from external environmental factors. It is also difficult to process its signal characteristics, which makes it difficult for its recognition accuracy to meet actual needs (Kim et al., 2018).

With the rise of big data and artificial intelligence technologies, deep learning algorithms have developed rapidly recently. Fruitful results have been achieved in the field of computer vision and speech recognition. Among them, the deep convolutional network represented by the CNN algorithm has low network complexity and strong feature extraction capabilities, which can well solve the problem of difficult feature extraction of EEG signals. Therefore, it is particularly urgent and feasible to recognize EEG signals based on deep convolutional networks.

## Related Research

The significance of studying brain-computer interface technology is not limited to promoting the development of rehabilitation medicine. More importantly, it opens up a new way for people to obtain brain information, and at the same time enriches the content of brain cognitive science and neuroinformatics. It has huge research prospects, important theoretical value and practical significance. As the basic research in brain-computer interface, the research of EEG signal recognition is a multidisciplinary problem (Aznan et al., 2016; Xu et al., 2020). The key lies in how to extract and classify EEG signals accurately and effectively. At present, many scholars have carried out in-depth research on this.

Most existing feature extraction methods rely on human knowledge and experience. However, due to the limitations of human knowledge and experience, artificially designed features have certain limitations, and they cannot extract suitable features well, resulting in limited classification accuracy (Wang and Bezerianos, 2017). In addition, the process of finding suitable features usually requires some additional experiments, which will take a lot of time and energy. Traditional EEG signal feature recognition research mostly uses Short Term Fourier Transform (STFT) or Wavelet Transform (WT) to extract the time-frequency features of EEG signals (Tabar and Halici, 2017; Lee and Choi, 2019; You et al., 2020). However, these tasks are generally based on the extraction of time-frequency features in a fixed time period of EEG data or in the same frequency band. There are limitations in extracting features of EEG data in fixed time bands and frequency bands. Based on the extracted time-frequency features, some researchers select features for different experimental subjects to improve the classification accuracy on motor imaging tasks. Luo et al. (2016) first uses wavelet packet decomposition technology to extract time-frequency features. Then use the dynamic frequency feature selection (DFFS) to select the feature with the highest classification accuracy for each experimental object. Li et al. (2017) first selects the time period with the highest correlation between the event-related desynchronization (ERD)/event-related synchronization (ERS) phenomenon in the collected EEG signals. Then use WPD to extract the time-frequency characteristics of the EEG signal. Finally, the feature selection algorithm is used to select the feature with the highest classification accuracy. Saa and Cetin (2012) proposed the Filter Bank Common Spatial Pattern (FBCSP). After filtering the original EEG signal with a set of filters, the CSP method is used to extract features on each filtered frequency band. Finally, the feature selection algorithm is applied on the basis of the extracted features. The above work research has improved the accuracy of some motor imaging tasks to a certain extent. However, since it takes a lot of time to select the characteristics of the experimental data set for each experimental object, it is not universal.

In recent years, deep learning has been widely used in computer vision, speech recognition and recommendation systems, and has achieved great success. Because the deep learning method can automatically extract the input signal features, it avoids the limitations of manual design features. Therefore, some scholars apply deep learning algorithms to the classification of motor imagery EEG signals (Chu et al., 2018; He et al., 2020). Among the many deep learning algorithms, Convolutional Neural Network (CNN) has become the most popular method in the motor imagination EEG classification algorithm because of its excellent feature extraction capabilities. Literature (Lawhern et al., 2018) proposed a CNN structure that can be applied to a variety of popular brain-computer interface paradigms (including motor imagination, P300 visual evoked potentials, etc.). A higher classification accuracy rate than the FB-CSP method is obtained. Li (2020) proposed a moving image classification algorithm based on spatiotemporal features extracted by convolutional neural network. The temporal and spatial characteristics of the EEG signal are extracted by the longitudinal convolution kernel and the lateral convolution kernel, respectively. And built a five-layer neural network model to classify EEG signals. Wei et al. (2019) uses EEG emotion data set SEED for emotion recognition research. The abstract features of EEG samples are automatically extracted based on the convolutional neural network in deep learning, eliminating the need for manual feature selection and dimensionality reduction. And with the most advanced methods at present, a considerable accuracy rate has been achieved. Li et al. (2020) proposed an algorithm combining continuous wavelet transform and simplified convolutional neural network to improve the recognition rate of MI-EEG signals. The feasibility of the algorithm is verified by the BCI dataset.

Drawing on the existing research work of EEG signal recognition, this paper proposes a motor imagery EEG signal recognition based on deep convolutional network. The main contributions are as follows:

1) Aiming at the difficult problem of EEG signal feature extraction, short-term Fourier transform is used to collect experimental data sets based on time series characteristics, so as to obtain EEG signals with time-frequency characteristics. And use continuous Morlet wavelet transform to further process the EEG signal difference of the data set. Provide high-quality data support for subsequent training and testing of deep convolutional network models.
2) It is oriented to the high accuracy and high precision requirements of EEG signal recognition for motor imagination. Based on the improved CNN network model, it realizes efficient recognition of EEG signals. Based on the advantages of the convolutional neural network’s own network model, combined with the CSP algorithm, two-level feature extraction and classification are performed on the motor imagination EEG signal. Further improve the quality of EEG signal feature acquisition. Ensure the efficient performance of EEG recognition for ideal data sets and measured data sets.

The rest of this article is organized as follows. The third section introduces the data set used in this article and explains the corresponding data preprocessing methods. The fourth section introduces the main principles of EEG signal recognition based on the improved CNN network model. The fifth section carries out corresponding experimental simulation analysis on the feasibility and optimality of the proposed method. The sixth section is the conclusion and outlook.

## Dataset Processing

### Sample Dataset

EEG signals are the distribution of potentials on the scalp produced by brain neuron activity, and are usually obtained by using an EEG device. The EEG data set used in this paper is BCI competiton data set.

The collection process of BCI competiton is described as follows: The subject wears an electrode cap and sits quietly in front of the computer, and imagines the movement of the left hand, right hand or right foot according to the prompts on the screen. Each subject performed a total of 280 motor imagination, 140 of which were left and right. The process of a single experiment lasting 7s is as follows:

(1) 1.5 s before the start of the experiment, a “++” prompt appeared on the screen to remind the subjects that they were about to perform the motor imagination task.
(2) At the beginning of the 1.5s of the experiment, the “+” disappeared, and L, R, F or arrows moving in different directions appeared in the center of the screen. According to the letters or moving arrows, the subjects imagined the movement of the left hand, right hand, and right foot. The process lasted 3.5 s.
(3) The arrows and letters on the screen disappear without any display. The subject can enter a relaxed state and rest for 2 s.

In the experiment, a 118-lead electrode cap was used to collect the EEG voltage on the scalp of the subject (Cheng et al., 2020). The acquisition frequency is 100 Hz. The EEG signals of all the above processes are collected by the system, so the EEG signals obtained are 5 matrices with 118 rows and 280^∗^7^∗^100 columns. Among them, the effective EEG signal of each subject performing the motor imaging task is a matrix of 118 rows and 280^∗^3.5^∗^100 columns.

**FIGURE 2:**
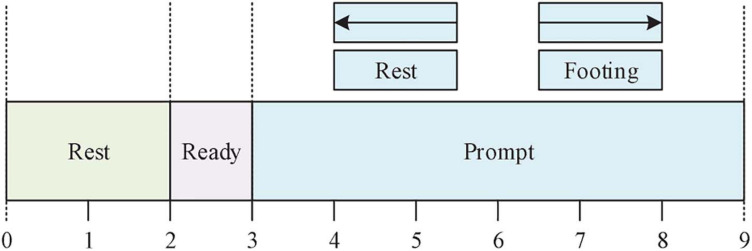
MI experimental paradigm.

During the experiment, the experimental paradigm shown in Figure 2 was used (Graimann et al., 2003).

### Data Preprocessing

When a person is performing limb motor imagination, a specific position in the motor sensory cortex of the brain will have regular potential changes (Pfurtscheller et al., 2005). When subjects perform unilateral limb motor imaging, the intensity of U rhythm (8–12 Hz) in the contralateral cortex of the brain decreases, and the intensity of Q rhythm (12–25 Hz) in the ipsilateral cortex increases, which is called event related desynchronization (ERD) and event related synchronization (ERS) phenomenon (Mousavi and Sa, 2019). These two phenomena are important basis for distinguishing different types of EEG signals. Therefore, the time-frequency domain analysis method that combines the two is one of the most efficient analysis methods (Padfield et al., 2019).

#### Short-Time Fourier Transform

The short-time Fourier transform first divides the entire time series into several time segments of equal length. Then calculate the frequency spectrum information in each time segment by Fourier transform. Obtain the change of each frequency component with respect to time from the surface. The calculation formula is as follows:

(1) $$S\,\left(f,k\right)=\sum_{n=0}^{N-1}s\,\left(n\right)\,\left[W\,\left(n-k\right)\,e^{\frac{f\,2\,\pi \,f\,n}{-N}}\right]$$

Where,*S*(*n*) represents the time series of EEG signals.*W*(*n*) represents window function.*N* represents the number of time points recorded.*k* represents the index of different time windows.*f* represents the frequency component in the signal.*n* represents time point. The length of the time window required to be divided in the formula is the same, which determines that the algorithm performs well when measuring high-frequency components. When measuring low-frequency components, it is often accompanied by distortion.

In order to effectively measure the change trend of the μ rhythm and β rhythm in the signal, this paper selects the time-frequency matrix obtained by the time window of 0.5s and the hamming window function (Soroosh and Mohammadi, 2018). Combine the time-frequency matrices on the two channels C3 and C4. A three-dimensional tensor with a size of 33^∗^35^∗^2 is obtained as the input of the subsequent convolutional neural network.

#### Continuous Morlet Wavelet Transform

The Morlet wavelet transform uses a wavelet of finite length and attenuation as the base to measure the intensity of each rate component in the signal over time. The formula is as follows:

(2) $$W\,\left(a,b\right)=\int_{-\infty }^{\infty }x\,\left(t\right)\,\frac{1}{\sqrt{a}}\,\psi \,\left(\frac{t-b}{a}\right)\,dt$$

Where,*x*(*t*) represents the signal sequence.ψ(*t*) represents the wavelet basis.*t* represents the time point. The parameter *a* controls the scaling of the wavelet function. When *a* takes a value from small to large, the wavelet function gradually widens, so the low-frequency components can be better measured. And by adjusting the parameter *b*, the shift of the wavelet function is controlled to obtain the intensity information of each frequency band at different time domain positions. The calculation formulas of Morlet wavelet center time and time domain span are as follows:

(3) $$\psi \,\left(x\right)=e^{-x^{2}}\,\cos\left(\pi \,\sqrt{\frac{2}{\ln2}}\,x\right)$$

(4) $$t_{0}=\frac{\int_{-\infty }^{\infty }t\,{\left|\psi \,\left(t\right)\right|}^{2}\,dt}{\int_{-\infty }^{\infty }{\left|\psi \,\left(t\right)\right|}^{2}\,dt}$$

(5) $$\Delta \,t_{\psi }=\sqrt{\frac{\int_{-\infty }^{\infty }{\left(t-t_{0}\right)}^{2}\,{\left|\psi \,\left(t\right)\right|}^{2}\,dt}{\int_{-\infty }^{\infty }{\left|\psi \,\left(t\right)\right|}^{2}\,dt}}$$

The calculation formula for center frequency and bandwidth is as follows:

(6) $$\omega_{0}=\sqrt{\frac{\int_{-\infty }^{\infty }\omega \,{\left|\Psi \,\left(\omega \right)\right|}^{2}\,d\omega }{\int_{-\infty }^{\infty }{\left|\Psi \,\left(\omega \right)\right|}^{2}\,d\omega }}$$

(7) $$\Delta \,\omega_{\psi }=\sqrt{\frac{\int_{-\infty }^{\infty }{\left(\omega -\omega_{0}\right)}^{2}\,{\left|\Psi \,\left(\omega \right)\right|}^{2}\,d\omega }{\int_{-\infty }^{\infty }{\left|\Psi \,\left(\omega \right)\right|}^{2}\,d\omega }}$$

Where, Ψ(ω) is the frequency component information obtained after ψ(*t*) undergoes Fourier transform. It can be known from the above formula that when the wavelet transform measures high frequency components, because the wavelet used is narrow, a smaller time domain span can be obtained, but the frequency domain span will be enlarged accordingly. Therefore, in the output time-frequency matrix, the resolution of the frequency dimension of the high frequency part is relatively low, and the low frequency part is just the opposite. Similarly, the C3 and C4 channel position information are integrated, and a sample matrix of size 35×1152×2 is obtained as the input of the neural network.

## Eeg Signal Classification Based on Improved CNN Network Model

### BCI Classifier Design and Training Process

The ability of deep learning algorithms to extract features is greatly improved compared with traditional algorithms. And generally, the more complex the network, the more sufficient features are extracted, and the better the result of the classifier. However, the advantages of the classification accuracy of deep learning algorithms are usually only reflected when the number of sample sets is large enough. And the more complex the network, the more parameters to be trained, the more training set samples will be needed.

**FIGURE 3:**
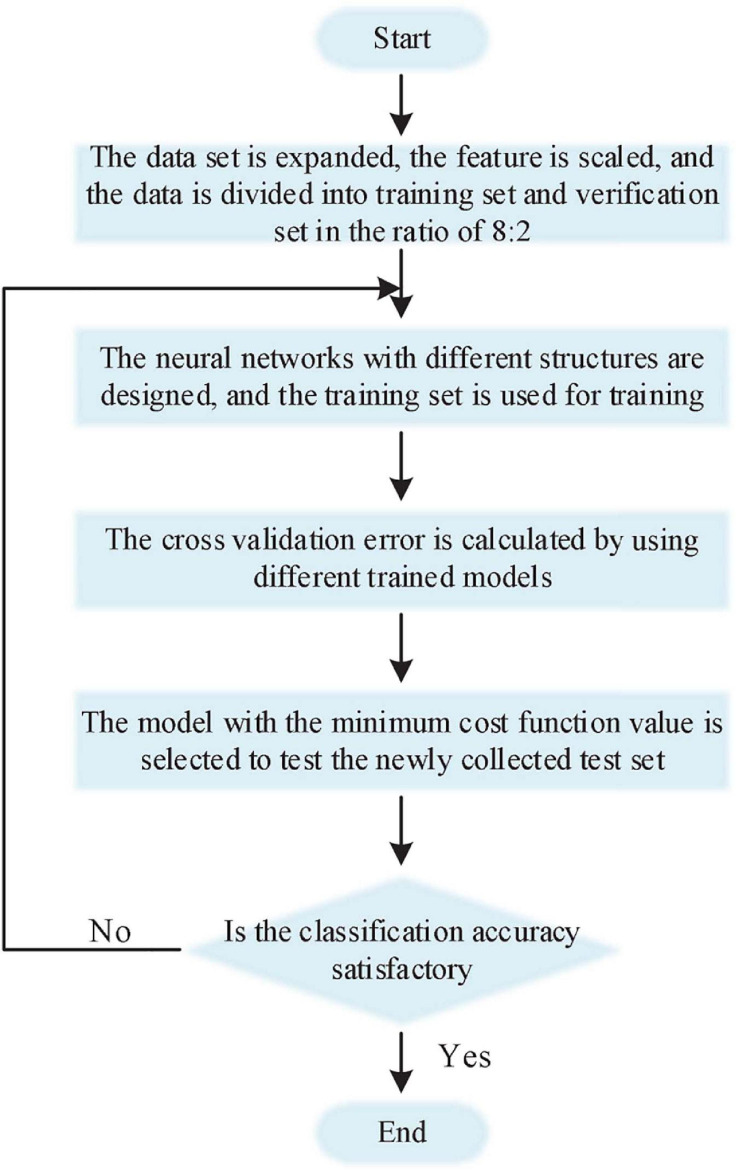
Flow chart of BCI classifier design and training.

It should be noted that the high complexity of the network model cannot be pursued blindly in the design of neural networks. We should balance the network structure and the number of sample sets, and design a preliminary neural network in advance (Anuse and Vyas, 2016; Ha and Jeong, 2019). Through the learning curve of the training set and the cross-validation set during the training process, it is judged whether the network is in an over-fitting or under-fitting state. Then debug the hyperparameters in the network according to the network status, and decide whether to increase the number of samples and how to modify the network structure. Until a network model with satisfactory classification effect is designed. Figure 3 is a flowchart of the brain-computer interface classifier design used in this article.

### Construction of Convolutional Neural Network

**FIGURE 4:**
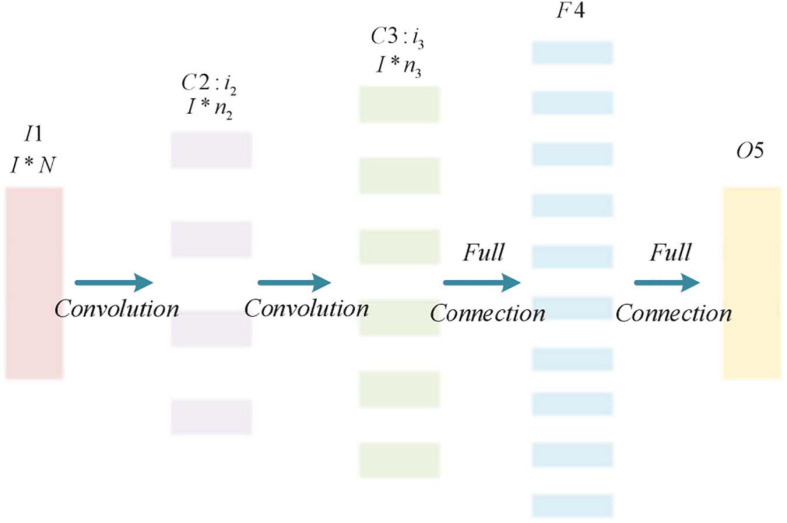
Improved CNN network structure.

In this paper, the classic CNN structure is modified, and the input sample data is reduced from the traditional two-dimensional to one-dimensional. The reconstructed CNN is used to extract and classify the one-dimensional feature data obtained after the motion imagination EEG is processed by the CSP algorithm. After the one-dimensional feature data of EEG is subjected to the feature extraction process again through the convolutional layer, the fully connected layer and the Softmax classifier are used to output the classification results. The CNN structure of this paper is shown in Figure 4.

The improved CNN structure is mainly divided into 5 layers, the first layer is the input layer (I1). The input sample data size is 1 × N. Among them, N is the number of features obtained after the motion image EEG is processed by the CSP algorithm, *N* = 4 × m; The second layer (C2) and the third layer (C3) are both convolutional layers for feature extraction of input sample data. The second layer (C2) has i2 convolution kernels, and the size of the convolution kernel is 1 × n2. The third layer (C3) has i3 convolution kernels with a size of 1 × n3. Due to the small length of the input sample data, the downsampling layer is omitted in this CNN; The fourth layer (F4) forms a single-layer perceptron together with the fifth layer (O5) in a fully connected way. After processing the output result of the third layer (C3), the classification result is output. The convolutional network is shown in Eq. 8.

(8) $$C\,N\,N=\left[I\,1\,\left(1\times N\right)-C\,2\,\left(i_{2}\times 1\times n_{2}\right)\right]-C\,3\,\left(i_{3}\times 1\times n_{3}\right)C\,N\,N=\left[I\,1\,\left(1\times N\right)-C\,2\,\left(i_{2}\times 1\times n_{2}\right)\right]-C\,3\,\left(i_{3}\times 1\times n_{3}\right)-F4-O5]-F4-O5]$$

It is assumed that the convolution kernel of each layer is represented by a matrix of size $\left[i_{1}^{*},\,n_{\text{l}}\right]$, where *i*_l_ represents the number of convolution kernels of the first convolution layer, and *n*_l_ represents the length of the one-dimensional convolution kernel. When training starts, these convolution kernels are initialized to random values between [-0.3, +0.3]. Then the error value Error between the network predicted category *Y*_i_ and the sample actual category *R*_i_ is corrected through the error back propagation algorithm, where Error is as in Eq. 9. If $W_{\text{i}}^{l\,\phi }$ and $b_{\text{i}}^{l\,\phi }$ represent the weight and deviation of the i-th convolution kernel of the l-th convolutional layer, the feature map $H_{\text{i}}^{l\,\phi }$ is as in Eq. 10. The input and output of the j-th neuron in the fourth (F4) and fifth (O5) layers of the fully connected layer are *x*^*l*^(*j*) and *y*^*l*^(*j*) respectively. The hyperbolic tangent function is used as the neuron activation function, and the connection method is shown in Eq. 11.

(9) $$E\,r\,r\,o\,r=\sum_{i=1}^{n}{\left(Y_{i}-R_{i}\right)}^{2}$$

(10) $$H_{i}^{l\,\phi }=H_{i}^{\left(l-1\right)\,\phi }\,W_{i}^{l\,\phi }+b_{i}^{l\,\phi }$$

(11) $$y^{l}\,\left(j\right)=\tanh\left[x^{l}\,\left(j\right)\,W^{l}+b^{l}\,\left(j\right)\right]$$

### Network Training

In the training process of the improved CNN classifier in this paper, there are still a large number of hyperparameters that need to be manually set. The specific hyperparameters related to network training in this experiment will be set as follows.

(1) Batch size and epoch: The batch size is the number of samples used in a training process. The value of the number of epochs is equal to all training samples divided by the batch size. In the training process of deep learning, all training sets are usually not loaded into memory at once for iterative calculations. Because the total number of training sets is too large, it will cause problems such as low memory efficiency and slow training speed. Experimenters need to consider the server memory size, input sample size, network model complexity and other factors to choose an appropriate batchsize. Make the network only read batchsize training samples during each training process. Setting the Batchsize too small will cause the network to be difficult to converge and underfit. If the batchsize is set too large, it will result in reduced efficiency or memory overflow. In this experiment, the batchsize is 64 and the epoch is 150.
(2) Learning rate: Learning rate is a very important hyperparameter in network training. Whether it is set reasonably can directly affect the final classification accuracy of the network. If the learning rate is set too small, the error curve will fall too slowly, and the learning rate will be too large, which will cause the error to explode, and the network cannot correctly find the direction of the gradient drop. After many attempts and comprehensive considerations, this experiment dynamically changes the learning rate during the training process. Set the initial value of the learning rate to 0.02 and the end value to 0.0002. Decay in the form of math.exp (- /decay_ speed). Where *i* is the number of iterations, decay_speed is the decay speed, and the value is 1,000 in this paper.
(3) Initialization of weights and biases: In this experiment, the weight *w*_i,j_ is initialized as normal random initialization, and the bias *w*_b_ is initialized as a constant matrix 0.1.
(4) Dropout:Overfitting often occurs in the training process of deep learning. Corresponding solutions include increasing the number of training set samples, adding a regularization function, and adopting methods such as Dropout. Since the convolutional neural network uses Relu as the activation function, the sparsity of the function makes the network self-regularized. Therefore, this experiment uses the Dropout method to set its value to 0.5, so that 50% of the hidden layer nodes do not work in the layer that uses Dropout during each training process. Thereby reducing the phenomenon of over-fitting and enhancing the expressive ability of the network model.

After designing the CNN network model and assigning all the hyperparameters in the model, start training the classifier. Convolutional networks are trained using BP algorithm (Jia et al., 2019; Liu et al., 2020), and the process is divided into three steps: The forward propagation calculates the output value *a*_ij_, the back propagation calculates the error δ_ij_, the gradient of the weight *w*_ij_ is calculated and the weight is updated.

The output value *a*_d,i,j_ of the forward propagation process of the convolutional layer is calculated as follows. Where*D*is the input depth and *F* is the size of the convolution kernel.*w*_d,m,n_ represents the weight of the *m*th row and *n*th column of the *d*-th convolution kernel.*x*_d,i,j_ represents the element in row *i* and column *j* of the *d* layer input.*f* is the Relu activation function.*w*_b_ is the bias of the convolution kernel.

(12) $$a_{d,i,j}=f\,\left(\sum_{d=0}^{D-1}\sum_{m=0}^{F-1}\sum_{n=0}^{F-1}w_{d,m,n}\,x_{d,i+m,j+n}+w_{b}\right)$$

Because the CNN network uses the connection method of the local receptive field and the down-sampling operation such as pooling processing, the calculation method of the error term of the model is very different from the traditional fully connected network. The derivation process of the *l-1*-th layer error term δ^*l*−1^ in the back propagation process of the CNN convolutional layer is shown in the following Eqs 13–16, where *E*_d_ represents the error function. The calculation of the error term of the CNN pooling layer depends on the specific pooling method. The error term of the maximum pooling is transferred from the next layer to the position corresponding to the upper layer $\delta_{i,j}^{l-1}$ intact, and the error term of the remaining positions is set to 0. The average pooling error term δ^*l*−1^ is evenly distributed from the next layer δ^*l*^ to each neuron corresponding to the previous layer.

(13) $$n\,e\,t^{l}=c\,o\,n\,v\,\left(W^{l},a^{l-1}\right)+w_{b}$$

(14) $$a_{i.j}^{l-1}=f^{l-1}\,\left(n\,e\,t_{i,j}^{l-1}\right)$$

(15) $$\begin{array}{c} \delta_{i,j}^{l-1}=\frac{\partial E_{d}}{\partial n\,e\,t_{i,j}^{l-1}}=\frac{\partial E_{d}}{\partial a_{i,j}^{l-1}}\,\frac{\partial a_{i,j}^{l-1}}{\partial n\,e\,t_{i,j}^{l-1}}\\ =\sum_{m}\sum_{n}w_{m,n}^{l}\,\delta_{i+m,j+n}^{l}\,f^{\prime }\,\left(n\,e\,t^{l-1}\right) \end{array}$$

(16) $$\delta^{l-1}=\sum_{d=0}^{D}\sum_{m}\sum_{n}w_{m,n}^{l}\,\delta_{i+m,j+n}^{l}\,f^{\prime }\,\left(n\,e\,t^{l-1}\right)$$

The last step of training is to calculate the gradient and update the weight accordingly. The pooling layer does not introduce the parameters to be learned, only the gradient of the weight and bias of the convolution layer needs to be calculated. Due to weight sharing, each weight *w*_i,j_ has an effect on each $n\,e\,t_{i,j}^{l}$, so its calculation formula is shown in the following Eqs 17 and 18. Finally, the network uses the gradient descent method to update the weights, as shown in Eq. 19. In the equation, η is the learning rate. Through repeated iterations of a large number of samples, the error function value is continuously reduced every time the weight is updated. Training of the CNN model.

(17) $$\begin{array}{c} \frac{\partial E_{d}}{\partial w_{i,j}}=\sum_{m}\sum_{n}\frac{\partial E_{d}}{\partial n\,e\,t_{m,n}^{l}}\,\frac{\partial n\,e\,t_{m,n}^{l}}{\partial w_{i,j}}\\ =\sum_{m}\sum_{n}\delta_{m,n}\,a_{i+m,j+n}^{l-1} \end{array}$$

(18) $$\frac{\partial E_{d}}{\partial w_{b}}=\sum_{m}\sum_{n}\delta_{m,n}^{l}$$

(19) $$w_{i,j}=w_{i,j}-\eta \,\frac{\partial E_{d}}{\partial w_{i,j}}$$

## Experiment and Analysis

In order to verify the feasibility and practicability of the method mentioned above, the experimental simulation robot hardware environment is: Lenovo Tinkpad E14, AMD Ryzen 7 4700U 8-core processor, 16GB memory, integrated graphics; The software environment is: operating system Chinese Windows 10, English version software Microsoft Visual Studio 2012.

This article uses the Caffe deep learning framework to implement model training and testing on the data set used in this article. The data samples in the experimental data set are divided into training set and cross-validation set at a ratio of 8:2, and 6,880 training set samples and 1,720 validation set samples are obtained. The deep convolutional network parameters are set according to the corresponding network model described in the section “Network Training.”

### Network Model Optimization and Analysis

#### The influence of Convolution Kernel Size on the Accuracy of EEG Classification

**FIGURE 5:**
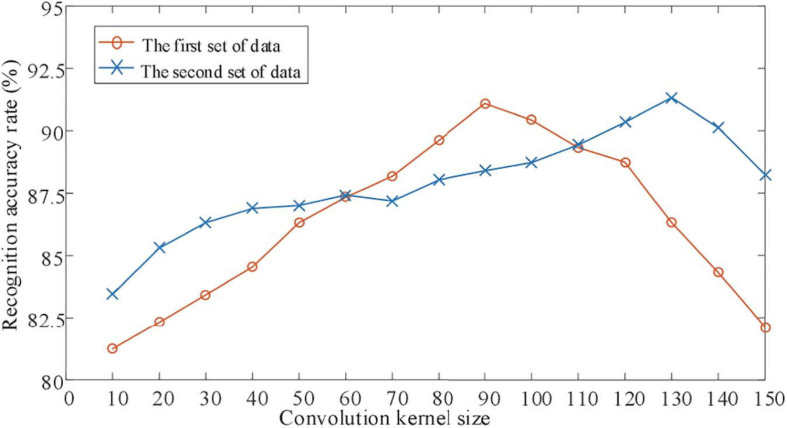
Influence of convolution check on identification results.

For the CNN network, determining the appropriate size of the convolution kernel is of great significance to the improvement of feature extraction and recognition accuracy. Therefore, for the classification of motor imagery EEG signals, this article first studied the influence of different convolution kernel scales on the identification of experimental data sets. The experimental results are shown in Figure 5.

It can be seen from Figure 5, where Figure 5 shows the changes in the classification accuracy of the motor imagination EEG signals of experimental objects as the size of the convolution kernel changes. It can be seen from Figure 5 that as the size of the convolution kernel increases, the classification accuracy of the EEG signal of the BBCI competiton dataset has just begun to gradually increase. Then gradually decrease, the optimal convolution kernel size is 1 × 115. The EEG signal classification accuracy rate of the measured data set increases first and then decreases as the size of the convolution kernel increases, but the optimal convolution kernel size is 1 × 55. This shows that for different experimental subjects, convolution kernels of different sizes are needed to extract the most suitable features for motor imagination brain electrical signal classification.

#### The Training Process of the Subject CNN Classifier

**FIGURE 6:**
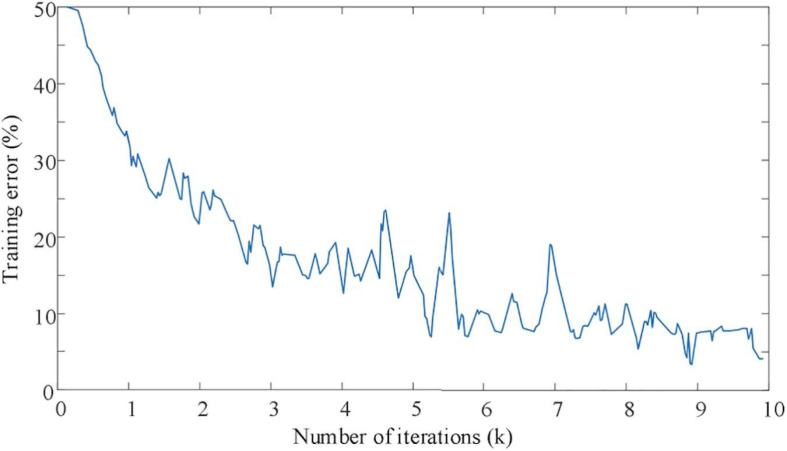
The curve of training error with iteration times.

**FIGURE 7:**
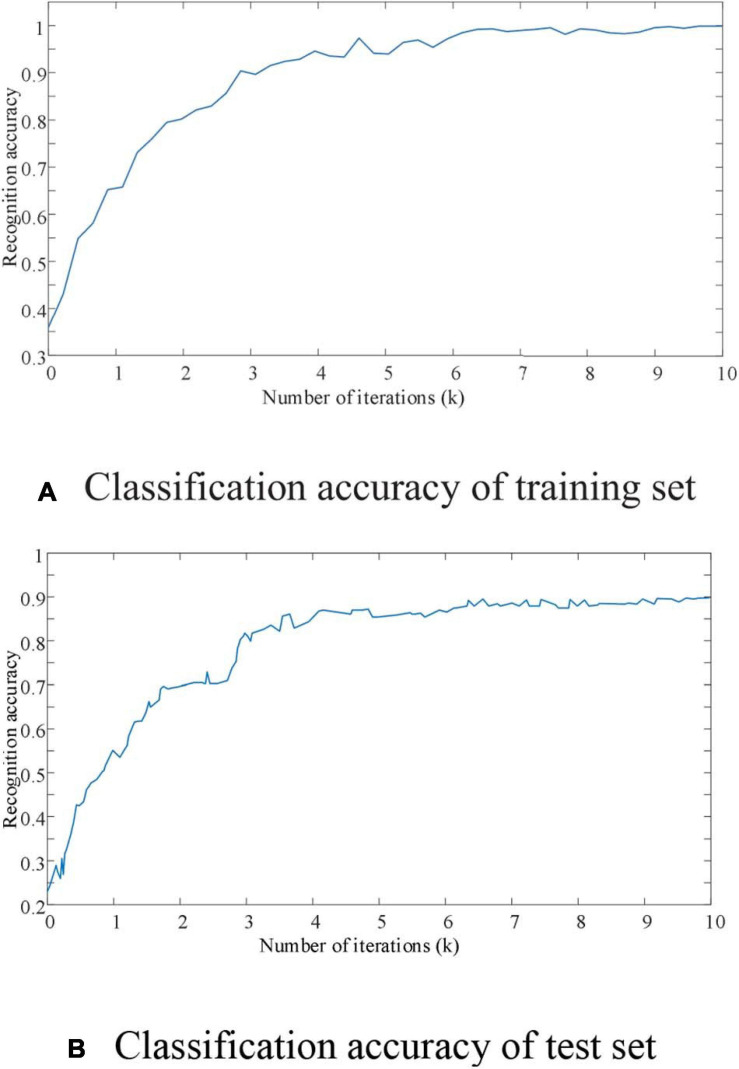
The curve of recognition accuracy with the number of iterations.

In order to further prove the feasibility and superiority of the method proposed in this paper for accurate identification of EEG signals, the experiment intends to analyze the training error and training accuracy of EEG signal identification under different iteration times. The experimental results are shown in Figures 6, 7.

As shown in Figure 6, it can be seen that the EEG signal data set is oriented to mixed motion imagination. Even though the training set data volume has high-dimensional and large-scale characteristics, the proposed improved CNN network model also shows excellent characteristics for the convergence speed of the recognition process. When the number of iterations of the improved CNN network reaches 8,000 times, the training error remains below 10%. At the same time, it can also be seen from Figure 7A that the improved CNN network has basically achieved full signal recognition after 9,000 iterations for the motion imaging training set data signal recognition accuracy used in this article. The identification of the sample test data set in Figure 7B also shows efficient convergence characteristics. That is to say, in 8,000 iterations, the recognition accuracy of EEG signals of motor imagination can achieve an effective recognition of more than 95%. Therefore, it is confirmed that the EEG signal recognition method proposed in this paper has the advantages of fast and efficient convergence characteristics.

### Identification and Analysis of Mixed Datasets

#### Analysis of Classification Results Based on Time Series

**FIGURE 8:**
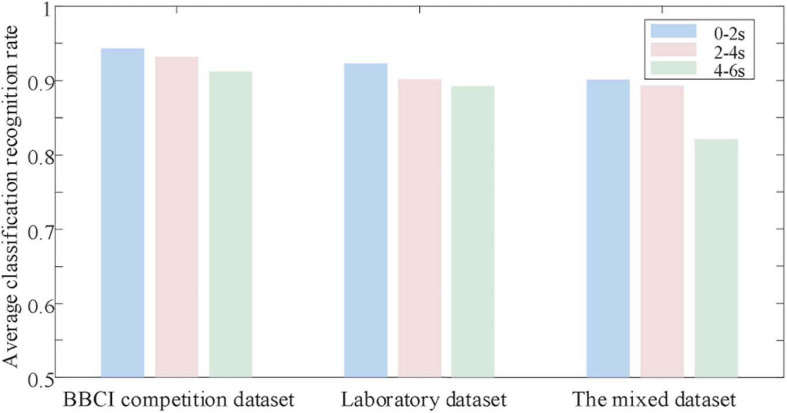
Classification results analysis based on temporal sequence.

The experiment divides the EEG experimental data of motor imagination into time periods, each with a duration of 2 s as input data. Figure 8 shows the average classification recognition rate of the tested users in the 3 time periods from 0 to 6 s.

It can be seen from Figure 8 that the average classification and recognition rate of EEG data in the first 2 s (0th–2nd s) is the highest. The average classification and recognition rate of EEG data in the last 4 s (2–6 s) is low. Explain that at the beginning of the experiment, the subject users focused on the motor imagination experiment. However, as time goes by, the concentration of the tested users decreases, which affects the quality of EEG data and ultimately leads to a decrease in the recognition rate. Therefore, according to the above analysis results, the original input data dimension is selected as 32 × 64, convolution and pooling 3-layer network, and the EEG data from 0 to 2 s after the start of motor imagination is selected for classification prediction.

#### Performance Comparison With Several Comparison Algorithms on Public Datasets

In order to verify the superiority of the deep convolutional network proposed in this paper in EEG signal recognition, this paper reproduced three other analysis methods based on the mixed motor imagery data set [Literature (Lawhern et al., 2018; Wei et al., 2019; Li, 2020)]. And get its evaluation index on the test set as shown in Table 1.

**TABLE 1:** Evaluation index of control models.

**Method**	**Accuracy**	**Discrimination**	**Sensitivity**	**Specificity**	**AUC**
The proposed method	0.9324	0.9653	0.8682	0.9243	0.9464
Lawhern et al. (2018)	0.9135	0.9211	0.8732	0.8932	0.9132
Li (2020)	0.8932	0.8976	0.8321	0.8589	0.8843
Wei et al. (2019)	0.8821	0.8832	0.7932	0.7932	0.8591

It can be seen from Table 1 that the accuracy index of the method proposed in this paper is 0.9324, which is higher than the literature (Lawhern et al., 2018) 0.0189, literature (Li, 2020) 0.0392, and literature (Wei et al., 2019) 0.0503. In terms of accuracy indicators, the improved CNN network is 0.0442, 0.0677, and 0.0821 higher than the comparison algorithm. As for the sensitivity index, the method proposed in this paper is not outstanding compared with the comparison algorithm, which is 0.005 lower than that in the literature (Lawhern et al., 2018). The fundamental reason is that the improved deep convolutional network has a deeper network depth, so that EEG signal recognition can guarantee higher accuracy during training and testing. But through the continuous learning and training of the deep network, this reduces the sensitivity of the network model to a certain extent. For the specificity index and AUC index, the methods proposed in this paper are 0.9243 and 0.9464, respectively, which are 0.0311 and 0.0332 higher than those in the literature (Lawhern et al., 2018).

## Conclusion

Motor imaging EEG signal recognition is an important and challenging research problem in human-computer interaction. Facing the accuracy and precision requirements of emotion recognition, this paper combines neural network and proposes a motor imagery EEG signal recognition method based on deep convolutional network. This method first uses short-time Fourier transform and continuous Morlet wavelet transform to preprocess the collected experimental data sets, so as to provide high-quality EEG signals for subsequent network models. Then, based on the improved CNN network model, the processed EEG signals are efficiently identified. Improve the quality of EEG signal feature acquisition and ensure the high accuracy and precision of EEG signal recognition. According to the analysis of the experimental results, the proposed method has an accuracy of 0.9324, an accuracy of 0.9653, and an AUC of 0.9464 for EEG signal recognition, and it has a good EEG signal recognition performance. The focus of future research will be to explore the platformization of the proposed method and strive to realize the commercialization of the proposed method. The focus of future research will be to explore the platformization of the proposed method and strive to realize the commercialization of the proposed method.

## Data Availability Statement

The original contributions presented in the study are included in the article/supplementary material, further inquiries can be directed to the corresponding author/s.

## Ethics Statement

Ethical review and approval was not required for the study on human participants in accordance with the local legislation and institutional requirements. Written informed consent for participation was not required for this study in accordance with the national legislation and the institutional requirements.

## Author Contributions

YF was proposed the main idea of this manuscript and completed the writing guidance, English polish and funding project. XX completed the algorithm design and experimental environment construction and completed the writing of the article. Both authors completed the experimental verification.

## Conflict of Interest

The authors declare that the research was conducted in the absence of any commercial or financial relationships that could be construed as a potential conflict of interest.
